# Association between Moderate-to-Severe Diarrhea in Young Children in the Global Enteric Multicenter Study (GEMS) and Types of Handwashing Materials Used by Caretakers in Mirzapur, Bangladesh

**DOI:** 10.4269/ajtmh.13-0509

**Published:** 2014-07-02

**Authors:** Kelly K. Baker, Fahmida Dil Farzana, Farzana Ferdous, Shahnawaz Ahmed, Sumon Kumar Das, A. S. G. Faruque, Dilruba Nasrin, Karen L. Kotloff, James P. Nataro, Krishnan Kolappaswamy, Myron M. Levine

**Affiliations:** Center for Vaccine Development, University of Maryland School of Medicine, Baltimore, Maryland; International Centre for Diarrhoeal Disease Research, Bangladesh (icddr,b), Dhaka, Bangladesh; University of Virginia, Pediatrics, Charlottesville, Virginia; Harlan Laboratories Inc., Indianapolis, Indiana

## Abstract

Handwashing practices among caretakers of case and control children < 5 years of age enrolled in the Global Enteric Multicenter Study in Mirzapur, Bangladesh were characterized and analyzed for association with moderate-to-severe diarrhea. Soap or detergent ownership was common, yet 48% of case and 47.7% of control caretakers also kept ashes for handwashing, including 36.8% of the wealthiest households. Soap, detergent, and ash were used for multiple hygiene purposes and were kept together at handwashing areas. Caretakers preferred soap for handwashing, but frequently relied on ash, or a detergent/ash mixture, as a low-cost alternative. Moderate-to-severe diarrhea was equally likely for children of caretakers who kept soap versus those who kept ash (matched OR = 0.91; 0.62–1.32). Contact with ash and water reduced concentrations of bacterial enteropathogens, without mechanical scrubbing. Thus, washing hands with ash is a prevalent behavior in Mirzapur and may help diminish transmission of diarrheal pathogens to children.

## Introduction

Diarrheal disease is estimated to be responsible for approximately one in ten deaths among children < 5 years of age globally, or a total of ∼800,000 fatalities annually.^1^ Water, sanitation, and hygiene (WASH) improvements have the potential to reduce rates of diarrheal disease by preventing exposure to infectious pathogens. Contaminated hands are one of the most common modes for transmitting human pathogens.^2^ Several meta-analyses of WASH interventions have suggested that investment in programs that promote handwashing with soap could generate a 30–48% reduction in the risk of diarrheal disease.^3^–^5^ However, the health benefits for investing in handwashing promotion depend upon successful and sustained uptake of good hygiene practices. Many handwashing programs have reported improvements in hygiene awareness, handwashing technique, and soap use; however, the evidence for sustained improvements in hygiene behavior is limited. The studies that have reported evidence of long-term behavior change coupled hygiene promotion with other interventions, making it difficult to attribute the sustained behavior uptake to particular factors.^6^–^8^ In contrast, other studies have suggested that the promotion of handwashing either had no impact or had an initial success with a subsequent decline in behavior to baseline frequencies at post-study follow-up visits after messaging had stopped and soap was no longer provided free.^9^,^10^

The cost of soap may be a particularly important impediment in achieving long-term improvements in handwashing. Poor households commonly report that soap is unaffordable, and allocate few resources for its purchase.^11^–^15^ Alcohol-based hand sanitizers that offer immediate disinfection without scrubbing have had some success in stimulating short-term use, however may face similar challenges with perceptions of cost among the very poor. In many communities, alternative materials, such as soil, mud, or ash, which are readily available without cost, are commonly used for various hygiene purposes, including washing hands and dishes. In Kolkata, India, 26% of rural villagers and 41% of slum residents reported use of ash for handwashing.^16^–^18^ In Bangladesh, 32.1% of urban and 55.5% of rural villagers reported using soil or mud for post-defecation handwashing, whereas 17.2% (urban) and 19.5% (rural) reported using ash.^19^ If the accessibility and affordability of ash trigger more frequent handwashing at critical times, promoting frequent handwashing with any material could then be effective at reducing the overall risk of transmission of infectious pathogens by contaminated hands.^12^,^20^,^21^

There is a paucity of data comparing health outcomes in households that use soap versus other types of low-cost, locally resourced materials. Our primary objective was to address this knowledge gap by analyzing whether the use of ashes for handwashing, instead of soap, by caretakers of children < 5 years of age enrolled in the Global Enteric Multicenter Study (GEMS)^22^ site in Mirzapur, Bangladesh was associated with a child's risk of developing moderate-to-severe diarrhea (MSD). Spot checks of household handwashing areas were used to rapidly and unobtrusively collect quantitative data on handwashing indicators. However, observed indicators may be poor proxies for complex handwashing behavior.^23^,^24^ To support the use of these spot-check indicators in the MSD study, our second objective was to conduct qualitative studies to characterize the use of hygiene materials among GEMS caretakers and confirm that observed materials were commonly used for handwashing purposes.

Finally, we explored the biological plausibility that exposure to ash and water for actual handwashing times could effectively reduce concentrations of enteric diarrheal pathogens that are common in this population.^25^ Microbiological evidence suggests that handwashing with ash or mud is as effective as soap at reducing the number of fecal coliforms on hands compared with washing with water alone or no washing.^16^,^19^,^26^–^28^ Ash in particular may possess antimicrobial properties that could reduce concentration of infectious pathogens on hands.^29^–^32^ We tested whether simple ash and water solutions could reduce concentrations of a spectrum of common human bacterial pathogens without scrubbing, and tested the functional role of pH in generating this effect.

## Materials and Methods

### The Global Enteric Multicenter Study.

Cases and controls were selected from among participants in GEMS.^22^ In brief, cases were children < 5 years of age living in the demographic surveillance system (DSS) area who visited Kumudini Women's Medical College and Hospital with MSD. Age, sex, and community-matched control children without diarrhea were randomly selected from the DSS database within 14 days of enrollment of the case.^22^ The MSD was defined as passing ≥ 3 loose stools within 24 hours, in conjunction with clinical signs of dehydration (sunken eyes, loss of skin turgor, or administration of intravenous fluids), dysentery, or admission to a health center or hospital.^33^,^34^ Demographic data were collected using a standardized questionnaire at enrollment from the caretakers of case children presenting at health facilities and at home for matched control children. Approximately 60 (range, 50–90) days after enrollment, a trained field worker visited case and control households to collect clinical and epidemiological data, and to perform spot observations of household WASH conditions (Table 1).^22^ An improved water source was defined based upon World Health Organization (WHO)/United Nations Children's Fund (UNICEF) Joint Monitoring Program (JMP) criteria, and where water was available daily and total collection time required < 30 minutes roundtrip.^35^ A handwashing station was defined as a designated area in the household with a source of water available. The observed presence of soap, detergent, ash, and a water source at the indicated household handwashing area served as the primary indicators of handwashing practice.

### Data management and statistical analysis.

Forms were scanned and sent electronically to the data coordinating center (Perry Point, MD) where the database was maintained.^36^ Epidemiological data were analyzed using SAS version 9.3 (SAS Institute, Inc., Cary, NC). Descriptive statistics were reported as proportions, means, and ranges. Means were compared using a two-sample *t* test. Categorical variables were compared using χ^2^. Reported *P* values are two-tailed; *P* < 0.05 was considered significant. A wealth index quintile (WIQ) was generated using principal component analysis of 13 household characteristics.^37^,^38^ Percent of case households owning the 13 household assets used for principal component analysis is shown in Supplemental Table 1. Conditional logistic regression was performed using data collected from matched case-control pairs to test for associations (shown as matched odds ratio, mOR) between handwashing indicators and MSD, while adjusting for wealth index quintile.^39^

### Qualitative studies.

We used qualitative data collection methods to gather more detail on hygiene practices of caretakers in our study, and to explore the perceptions and determinants related to the selective use of different types of handwashing materials. Qualitative approaches included focus group discussions (FGDs) conducted in the community, semi-structured in-depth interviews (IDIs) with mothers or caretakers, and key informant interviews (KIIs) of grandmothers, conducted in the home of young children enrolled in GEMS (Table 1). Caretakers were selected for participation in these qualitative studies from the database of GEMS cases and controls that had already completed the follow-up visits in the case-control study (Table 1). Caretakers were selected to achieve diversity in access to a sanitation facility, which included caretakers who shared a facility with other families, owned a private facility, or had no access whatsoever. Participants were contacted by cell phone and FGDs, IDIs, and KIIs were conducted with those who expressed their willingness to participate.

A manual for FGD, IDIs, and KIIs was developed based upon research objectives, translated into Bengali, back-translated, pilot-tested, and refined before data collection. Topics that were explored included types of personal and household hygiene materials that caretakers use, where they were acquired and stored, when and how they use them, and the motivations for using various types of cleaning materials. The FGD sessions were conducted in the community, with each session lasting 40–45 minutes. We conducted nine FGD sessions (group size of 6–8 participants), which included 26 case caretakers and 25 control caretakers. Twelve caretakers (6 cases, 6 controls) underwent IDIs and 12 grandmothers (6 cases, 6 controls) who had not previously participated in a FGD took part in a KII; these sessions lasted for about 30–40 minutes. All interviews and group discussions were conducted in Bengali. Two research officers (authors FDF and FF) with an educational background in Nutrition and Public Health and professional training and experience in qualitative research methodology carried out these sessions and interviews. FGDs, IDIs, and KIIs were recorded using audio-tape, which was then transcribed verbatim and translated into English.

### Analysis of qualitative data.

Data analysis of transcribed audiotapes involved extensive memoing to identify core inductive themes that were then used to manually code the entire data set according to our research objectives, and generate code-specific reports for detailed analysis. After coding, we translated these data into English and compared for consistency. The KIIs, IDIs, and FGD results were analyzed individually to verify consistency in the content of caretakers' responses in group and private interviews, and then compared as one body of information for drawing inferences for descriptive reporting. We performed thematic content analysis to provide a descriptive narrative of our results. No attempt was made to quantitatively compare study findings.

To document the process that caretakers use for handwashing, the first 10 case and control caretakers who were being visited for a 60-day follow-up visit were asked to demonstrate their technique. An observer recorded the steps used by each caretaker, and estimated the amount of ash and water used. A stop watch was used to time how long the caretaker scrubbed her hands after wetting hands with water or a handwashing material.

### Microbiology.

The caretakers who performed handwashing demonstrations were asked to describe where they obtained the ash used for handwashing and to provide a sample of ash from their designated container in the handwashing area, which was then sent to the Center for Vaccine Development (CVD), University of Maryland, Baltimore, MD. A series of experiments was conducted to assess the ability of specific bacteria to survive in ash as follows. Before the laboratory experiments, ashes were autoclave-sterilized. Fresh bacteria for spiking experiments were isolated by streaking swabs from glycerol stocks maintained in the CVD repository onto selective media and culturing plates overnight at 37°C. *Aeromonas hydrophila* was cultured on Ryan medium, *Vibrio cholerae* O1 on thiosulfate citrate biosalts (TCBS) agar and *Shigella flexneri* 2a, enteraggregative *Escherichia coli* (EAEC) and enterotoxigenic *E. coli* (ETEC) on MacConkey's agar. Survival of each bacterial strain in ash and water was determined by spiking concentrations of each bacteria type in triplicate into slurries of sterile water and ash collected from one of three different Mirzapur households chosen randomly from the 10 samples. Slurries of ash and water were mixed at a 1 gram autoclaved ash per 5 mL autoclaved water ratio to replicate crude observed amounts demonstrated by Mirzapur caretakers. The pH for each ash and water slurry was tested using pH test strips (Sigma, St. Louis, MO).

Bacterial inocula (optical density [OD] 600 = 1.0) were prepared by collecting swabs of bacteria from fresh overnight cultures into phosphate buffered saline. Concentrations of inoculating solutions were confirmed by plating 10-fold serial dilutions on appropriate selective media. For each experiment, 10^4^ colony-forming units (CFU) were spiked into each of the three separate tubes with 2 grams ash and 10 mL water, and mixed by rotating the tube five times (spiking inoculum concentration of 10^3^/100 uL). One tube of each of the three ash samples was spiked with 100 μL of water as a negative control. After 30 seconds and 1 minute time intervals, 100 μL of spiked ash slurry were plated in duplicate upon an appropriate culture medium. After overnight incubation at 37°C, the total number of recoverable CFU on duplicate plates were counted and averaged. The concentration of replicate ash samples was consistent, therefore the results for each of the three ash samples types were averaged to report percent reduction and log reductions in bacteria. Percent reduction is reported as CFU remaining per original spiking concentration. The role of pH in bacterial killing was explored by preparing ash and water slurries, and adding HCl to neutralize the pH to 7.0. Spiking experiments were repeated and recovered CFU quantified.

### Ethical considerations.

Written informed consent was obtained from adult caretakers of children enrolled in the GEMS case-control study.^22^ Consent for the qualitative studies involved describing the research objectives and methods to participants before each and every session. Written consent was obtained indicating agreement to participate and willingness to have the session audio taped. Study protocols were approved by the Research Review Committee and Ethical Review Committee of the International Center for Diarrheal Disease Research, Bangladesh (icddr,b) and the University of Maryland Baltimore Institutional Review Boards.

## Results

### Socio-demographic characteristics of matched case-control pairs in Mirzapur.

A total of 1,394 case and 2,465 control children were enrolled between December 2, 2007 and December 5, 2010 in the GEMS case-control study in Mirzapur, Bangladesh.^40^ Case and control children lived in similar household environments (Table 2). Burnable materials such as grass, agricultural residue, and animal dung accounted for more than 95% of the fuel sources used by caretakers, which is consistent with 2009 surveillance data in Bangladesh (Table 2).^41^ Nearly all case (99.4%) and control (99.3%) caretakers used an improved deep or shallow tube well water source as their primary source of drinking water (Table 2).

### Handwashing indicators in households of matched case-control children in Mirzapur.

Household spot checks were used to observe and record all indicators of hygienic behavior in a total of 1,374 case and 2,429 control households. A designated handwashing area with water was observed in 99.2% of case and control households, and a handwashing material such as soap, detergent, or ash was observed in almost all households at that location (Table 3). Soap or detergent was observed at handwashing locations in 88.4% of case and 87.8% of control households, and a container of ashes was observed in 48.0% cases versus 47.7% controls (Table 3). Just over half of the cases (51.3%) and control caretakers (51.7%) had only soap or detergent, and 10.9% of case and 11.6% of control caretakers had only ash (Table 3). Children in households with only soap or detergent were just as likely to be cases as children in households that had ash only (matched odds ratio [mOR] = 0.91; 0.63–1.32, Table 3).

### Relationship between indicators of handwashing practices and wealth.

Although wealth quintile was not associated with risk of MSD, it was strongly associated with the type of hand cleanser available at the household's handwashing station. Ownership of soap or detergent significantly increased from 78.6% of the poorest households to 98.6% of the wealthiest households (*P* < 0.0001, Table 4). Use of only soap or detergent was also statistically more common among the wealthy (63.0%) than the poor (45.3%). Ownership of ash showed the opposite trend, progressively decreasing with wealth quintile from 52.0% of the poorest households to 36.8% of the wealthiest (*P* < 0.0001). Having a container of only ash for handwashing, with no soap or detergent, has decreased with wealth quintile from 18.8% of the poorest households to 5.7% of the wealthiest (*P* < 0.0001). Although soap and ash ownership showed significant patterns of association with wealth of a household, there was no difference between case and control caretakers in ownership of any soap (*P* = 0.85), soap only (*P* = 0.98), any ash (*P* = 0.50), or ash only (*P* = 0.10) (Table 4).

### Qualitative studies on the uses of hygiene materials found in Mirzapur households.

Qualitative studies were used to understand how and when caretakers choose to use specific types of hygiene materials, especially for handwashing, how those materials are obtained and stored, and the motivations for using non-soap materials for handwashing.

#### i. Use of hygiene materials at handwashing areas in Mirzapur households.

Caretakers use a variety of materials for multiple personal and household purposes. Ash and detergent serve many household needs, such as for laundry (detergent only), dishwashing, and cleaning and sanitizing the latrine (both) (Table 5). The *Pucca* toilet users (of both shared and privately owned facilities) stated that cleaning the toilet with ashes prevents bad odor. Ash, along with a broom, is considered to be a suitable substitute for toilet cleaner products. However, ash has additional uses for caretakers using a pit latrine and practicing open defecation. Caretakers using a pit latrine reported that facilities frequently look and smell bad, so they spread ash in the latrine pit every morning to minimize the smell. They also frequently observe insects on the feces; chickens and hens roam around the household and eat the insects (and feces). Therefore, ash is also spread in the latrine to prevent “*growing*” these insects. Caretakers who reported having no access to a facility, or who had a facility but couldn't conveniently use it at that time (such as in an emergency), would go to an open area (jungle or river side) for open defecation and use ash to cover their feces to prevent the smell.

Caretakers have a high degree of personal hygiene awareness, and understand that lack of hygiene is related to diseases like diarrhea. The action of handwashing at specific times with any material is considered a priority.

“We have to maintain our children and feed him; it's better if our hands are clean at that time.”

“It (handwashing) matters a lot; it affects our body. It causes diarrhea, dysentery, etc.”

“After using toilet, our hands might be contaminated with germs and soap can clean those germs; for that cleaning purpose, we use soap.”

They reported that soap is the only material used for bathing one's self or a child, and is the primary material for handwashing after defecation, cleaning a child, before feeding a child, and at any point where she felt she had contacted a dirty thing (Table 5). However, detergent powder, ash, a mixture of ash and detergent, and soil from the area outside the latrine or in the yard are all commonly used for handwashing as well, particularly after defecation or after cleaning a child who defecated (Table 5). The following quotes illustrate the diversity in hand cleaning practices, and the pervasive use of words like “sometimes” and “occasionally” that reveal inconsistent use of particular materials:

“Sometimes, I rub my hands on the soil.”

“Soap sometimes; ash occasionally.”

“I use soil (for hands) after cleaning the baby's bottom; sometimes soap is used.”

Furthermore, handwashing is not always performed with one single material or mixture of materials. Some caretakers indicated a multistep process where a second material was used sequentially to wash if the hands were still perceived to be dirty or malodorous.

“As I use ash first (after defecation), I think, it might not clean my hand properly, for that reason I use two agents (soap later) to clean my hands properly.”

#### ii. Access, procurement, and storage of household hygiene materials.

Caretakers indicated that they purchase soap and detergent using household income from a local vendor, and store these materials beside the household tube well or outside the latrine where hands are usually washed (Table 5). Sometimes soap is kept inside the toilet. Ash is obtained within the household as a byproduct from cooking fires. Caretakers primarily use earthen burners (made of soil) for cooking and fuel the fire with logs, branches, leaves, straw, shrubs, sticks, bamboo, and dried cow dung. The ash obtained from burning these materials is considered quickly and easily available and is a low-cost resource that can be recycled for many household purposes. Ash is also stored in pots outside the latrine and at the tube well (Table 5). Soil is not kept in a container that can be observed during household spot checks; in this case, hands are typically rubbed on the ground.

#### iii. Handwashing technique.

Caretakers participating in handwashing demonstrations first grabbed a handful of ash, or an ash and detergent mixture (10–15 grams), from a pot in their designated handwashing area. They wet their hands and the ash with water from a pitcher or faucet (mean volume ∼50 mL), scrubbed their hands for 15 to 30 seconds, and then rinsed with 0.75–1.0 L of water.

To understand whether caretakers made distinctions between the need to wash the left and right hand, we asked the mothers to describe when they felt that they needed to wash both of their hands. One mother reported washing her (right) hand with water only before feeding the child. Caretakers listed times when both hands were washed as during cleaning utensils, handling cow dung, after defecation, after completion of household tasks, before cooking, before eating, and after cleaning the bottom of the child (that defecated).

#### iv. Motivations for handwashing (or not washing) with specific cleaning materials.

Quantitative and qualitative studies clearly show that caretakers perceived soap as the best way for washing hands, so we asked them to describe the situations where they might not wash at all, or wash with ash, soil, or water only instead of soap. The primary factors that influenced their practices included: perceived necessity, stress, convenience, affordability, and environment.

Perceived necessity influenced whether a caretaker washed at all with any material. Some caretakers did not think washing hands after defecation was necessary if they were going to perform some other task involving detergent or detergent/ash.

“I use detergent powder to clean my hand; but if I have to clean my utensils after defecation, that time I do not wash my hands because I wash crockeries and utensils with soap; so, I do not need to clean my hands.”

Convenience of access influenced the type of material caretakers used for washing. Ash and detergent seemed to be more consistently available within the household. Caretakers pointed out that sometimes a bar soap was finished before she noticed, and was not available when needed. If she was busy with household chores, usually perceived to be the case, she would collect detergent or ash from close to the household, instead of going to purchase another bar at that time. Convenience of time influenced whether caretakers washed at all, and the type of material they washed with. Caretakers felt that they were frequently too busy to wash their hands, and suggested that washing with water only or ash was quicker and easier. Some critical times, such as feeding a child, were prioritized as important times to wash, regardless of time constraints.

“We have to work a lot, sometimes we get time to clean our hands and sometimes not: usually we clean our hand with water before eating and feeding our child; all of us do the same practice.”

“Sometimes it happens that we couldn't apply soap due to time constraint; I just wash my hands with water or with ash sometimes.”

Caretakers emphasized that the cost of soap was a major limitation, and that they could not afford to use it at all times for all purposes. Thus, they used alternative materials that performed equally well and come at lower cost.

“Soil is also used; who can use so many soaps?”

Environmental factors also played an influence on material use. Caretakers informed us that this area had iron in the water supply, so ash and detergent are mixed to produce a better cleaning product than with either material alone.

### Survival of enteric bacteria exposed to liquid ash.

**Figure 1.**
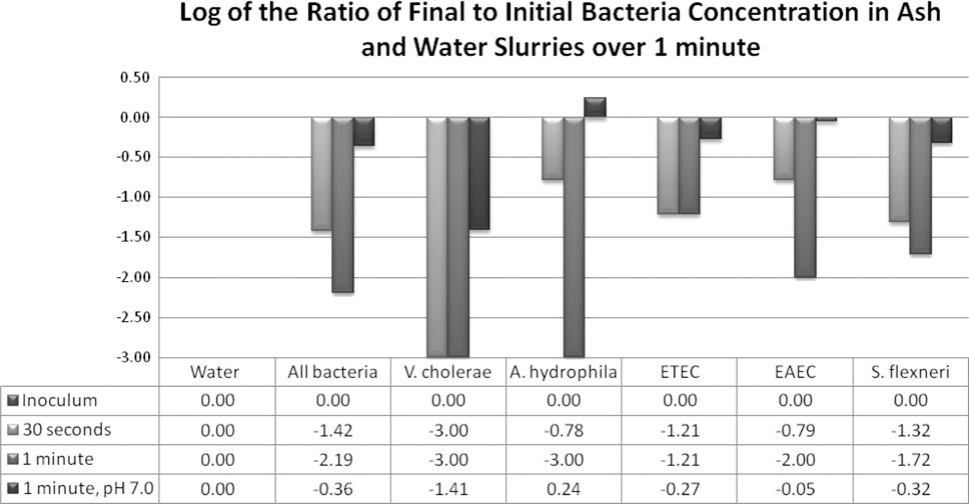
Log of the ratio of the final to the initial concentration of enteric bacterial pathogens at 30 seconds and 1 minute time points after exposure to slurries of ash (pH = 10.5) used for handwashing in households in Mirzapur, Bangladesh, and to slurries of ash at neutral pH.* Negative values indicate reductions in concentration of detected bacteria. All values are means of triplicate experiments using three different sources of household ash and initial spiking inoculums of 1,000 cfu/100 uL.

To simulate, in the laboratory, the ability of several bacterial pathogens to persist in a mixture of ash and water, we inoculated known quantities of each bacterial strain into a 1:5 mixture of ash and water (pH range 9.5–11.5) for set time intervals. After 30 seconds, no *V. cholerae* were detected using selective media (Figure 1). After 30 seconds of exposure to ash and water, the log of the ratio of the final to the initial concentration was −1.3 for *S. flexneri* (95% decrease in concentration), −0.8 for EAEC (83.8%), −1.2 for ETEC (93.9%), and −0.8 for *A. hydrophila* (82.4%) (Figure 1). After 1 minute of exposure to ash and water, the log of the ratio of the final to the initial concentration was −1.9 for *S. flexneri* (98.9%), −2.0 for EAEC (99.0%), −1.2 for ETEC (93.9%), and −2.6 for *A. hydrophila* (99.7%) (Figure 1). No reduction in bacterial concentrations was observed when the pH slurry was adjusted to 7.0 (not shown).

## Discussion

The primary objective of this study was to analyze whether hygiene practices of GEMS case-control caretakers were associated with moderate-to-severe diarrhea in children. We used household spot checks to efficiently quantify the presence of handwashing materials in GEMS households. Spot checks of handwashing stations with water and soap are commonly used as a proxy for handwashing practices in demographic health surveys and studies,^9^,^10^,^42^–^44^ Evidence of association between the presence of water and soap in a handwashing area and observations of improved handwashing behavior is mixed.^23^,^24^,^41^ In one study in Bangladesh, the presence of water and soap doubled the probability that the subject would be observed washing both hands with soap.^41^ Additionally, the presence of soap at a handwashing station has been associated with significantly cleaner hands of household members.^45^ However, concordance between observed behaviors and environmental conditions can be variable within households over repeat visits, showing that behaviors may be consistent but not absolute.^24^ Furthermore, as our qualitative studies showed, materials like ash and detergent can be used for multiple personal and household purposes, and their presence at a handwashing area may be the result of use for toilet maintenance as much as for handwashing.

We used a mixed-method approach of quantitative household spot checks for hygiene indicators and qualitative handwashing demonstrations, interviews, and focus group discussions to characterize handwashing practices among GEMS caretakers and confirm that spot check indicators were valid proxies for handwashing practices. Household spot checks documented that nearly all households with young children maintained a designated handwashing area equipped with a source of water and a type of cleansing material. Ash, soap, and detergent were all common handwashing and household cleansing materials, and were frequently stored together at handwashing stations. The use of ash for post-defecation handwashing has been previously reported as a common practice in rural and urban slum areas of Bangladesh, and in urban Kolkata, India.^17^,^19^ Our qualitative and observational data shows that despite caretakers reporting primary use of soap, many of those caretakers experience constant (environment, poverty) and dynamic (type of critical event, convenience of time) pressures that motivate them to frequently wash with ash after defecation instead of soap. These studies revealed underlying variability and complexity in the use of household cleansing materials for handwashing, and showed that spot checks failed to capture the less overt practices of mixing ash and detergent and scrubbing hands with soil from the ground. However, they also confirmed that observed soap/detergent and ash are a valid proxy for primary post-defecation handwashing practices in this population.

There are few studies that have assessed the efficacy of washing with ash on risk of diarrhea. In one instance, a lower risk of diarrhea was observed for children whose mothers washed their hands with soap after defecation versus with water only, but was not observed for children of mothers who washed with ash.^46^ In another study, there was no difference in diarrhea in children if mothers washed with any material, including ash, soap, and soil versus did not wash their hands or washed with water only.^47^ We did not find an association between having a designated handwashing station equipped with a source of water and a type of handwashing material and MSD in children. However, these results must be interpreted with caution, because the size of the reference group (households who owned no hygiene material) was extremely low (< 1%). More importantly, rates of MSD were similar for children in households who owned only ash versus children in households who owned only soap or detergent, suggesting that washing with ash can be equally effective at preventing exposure to unclean hands as soap. To our knowledge, this is the first study to compare health outcomes in households using soap versus alternative materials like ash.

We tested whether simple contact with ash and water for typical handwashing times could reduce the risk of hand-transmitted disease by decreasing concentrations of enteric pathogens that were common causes of MSD in our Bangladesh study.^40^ It has been proposed that using ash, mud, or clay is less desirable than soap because those materials could harbor and transmit contaminating bacteria.^48^ However, previous handwashing experiments showed the opposite, showing that mechanically scrubbing hands with ash, soil, and soap similarly reduced levels of fecal coliforms on hands (95–96% ash, 93% soil, 90–92% soap).^27^,^28^ Additionally, ash is very effective at pathogen inactivation in sewage and sullage.^29^,^49^ Our data expands upon these studies by showing that alkaline ash slurries reduce concentrations of common diarrheal bacterial pathogens by two or more orders of magnitude within a typical 30-second handwashing event.^32^,^50^,^51^ Ash is likely bactericidal, although it could also cause co-precipitation of bacteria with ash particles.

One caveat of this study is that household spot checks for handwashing materials were conducted roughly 2 months after the clinically confirmed episode of diarrhea in a child. It is possible that case and control children might have experienced diarrhea in that window of time or that caretakers switched handwashing materials. Additionally, we did not use methods to assess whether the spot checks were a valid proxy for *frequency* of handwashing behavior among caretakers in this population. Studies employing motion-tracking sensors embedded in soap have found poor correlation between soap use and proxies like the presence of a handwashing area, water, and soap.^52^,^53^ Although case and control caretakers owned similar levels of soap/detergent and ash, the actual frequency and timing of handwashing could still be significantly different. Variation in actual behavior is most likely the greatest influence on exposure to infectious enteric pathogens for children, yet is the most challenging to accurately measure.

Ultimately, promoting handwashing at critical times with any locally available resource may be effective for achieving sustainable improvements in handwashing behavior.^12^,^54^ Providing free soap with hygiene education has been very successful at improving hygiene awareness, but has produced mixed results in actual short-term and long-term behavior formation.^9^,^13^,^53^ Once households are left to assume the financial burden for providing soap for themselves, soap use can revert to baseline levels.^9^,^13^ In areas where ash is already commonly used, readily available, and a cost-free resource, effective messaging that promotes more frequent use of scrubbing hands at critical times, especially with ash, would reduce non-compliance caused by perceptions of cost and acceptability. This strategy may also be successful in areas where ash and detergent are combined to improve washing with hard water. Promoting more frequent and timely use of soap or ash will likely be effective among all wealth classes in Mirzapur.

Although the poorest households are the most likely to use ash, 36.2% of the wealthiest households in Mirzapur still keep ash at a handwashing area.^27^,^46^ This reflects some motivation at all wealth levels to resourcefully balance the use of soap or detergent supplies with other no-cost materials for household needs. Finally, the convenience of access to particular materials and the caretaker's perception of time availability had a significant influence on both the type of material used for washing and on whether washing occurred with any material. Hygiene promotion in this community should stress that frequent and effective handwashing behavior is a priority, and help them create enabling and accessible environments that encourage handwashing even when they are rushed.

## Supplementary Material

Supplemental Table.

## Figures and Tables

**Table 1:** Types of data collected*

Method of data collection	Size of sample	Selection
Interviews with caretakers of young children	3,859 Caretakers (1,394 cases, 2,465 controls)	Cases presenting with moderate-to-severe diarrhea at health center, age- and sex-matched controls selected from community
Household spot checks during follow-up visits	3,803 Caretakers (1,375 cases, 2,428 controls)	Cases presenting with moderate-to-severe diarrhea at health center, age- and sex-matched controls selected from community
Focus group discussion	9 FGDs (26 case, 25 control)	Participants were case and control caretakers who had already completed follow-up visit, and were selected to represent diversity in sanitation access
In-Depth Interviews with caretakers of young children	12 IDIs (6 case, 6 control)	Participants were case and control caretakers who had already completed follow-up visit, and were selected to represent diversity in sanitation access
Key Informant Interviews with caretakers of young children	12 KIIs (6 case, 6 control)	Grandmothers of case and control children who had completed follow-up visit, but had not participated in FGD or IDI
Handwashing demonstrations and sample collection for bacteriology	10 Households	10 successive case and control caretakers scheduled for follow-up visit in March 2011

* FGD = focus group discussions; IDIs = in-depth interviews; KIIs = key informant interviews.

**Table 2:** Socio-demographic characteristics and the univariable odds of moderate and severe diarrhea collected at enrollment from caretakers of GEMS-matched case and control children between 2008 and 2010 in Mirzapur, Bangladesh

	Case, *N* = 1394	Control, *N* = 2,465	mOR (95% CI)	*P*
Male child	803 (58.4%)	1401 (57.7%)	−	0.69
Mean age:				
0 to 11 months, *N* = 1,409	7.2 (2.6)	7.0 (2.6)	−	0.19
12 to 23 months, *N* = 1,216	16.7 (3.3)	16.7 (3.1)	−	0.87
24 to 59 months, *N* = 1,178	35.0 (8.6)	34.9 (8.6)	−	0.85
Mean people in household	5.8 (2.8)	5.8 (2.8)	−	0.97
More than 1 child < 5 years of age in household	403 (29.3%)	711 (29.3%)	0.97 (0.84–1.13)	0.70
Both parents live in home	950 (69.1%)	1786 (72.8%)	0.82 (0.71–0.96)	0.01
Caretaker's education:				
None or some primary	345 (25.1%)	605 (24.9%)	Ref.	Ref.
Completed primary	891 (64.8%)	1,585 (65.3%)	1.06 (0.90–1.25)	0.47
Beyond primary	139 (10.1%)	238 (9.8%)	1.05 (0.82–1.36)	0.69
Cooking fuel (any):				
Electricity/Propane/Gas	84 (6.1%)	138 (5.7%)	1.10 (0.79–1.52)	0.59
Wood, Grass, crop residue	1,308 (95.1%)	2,324 (95.7%)	0.92 (0.76–1.11)	0.37
Animal dung	850 (61.8%)	1,527 (62.9%)	0.95 (0.81–1.10)	0.39
Wealth index quintile:				
1 (poorest)	286 (20.8%)	473 (19.5%)	Ref.	Ref.
2	267 (19.4%)	490 (20.2%)	1.02 (0.82–1.27)	0.84
3	275 (20.0%)	474 (19.5%)	1.04 (0.84–1.30)	0.70
4	283 (20.6%)	493 (20.3%)	1.05 (0.85–1.30)	0.65
5 (wealthiest)	264 (19.2%)	498 (20.5%)	0.97 (0.78–1.21)	0.79
Improved drinking water source (requiring < 30 minutes to fetch)	1,385 (99.4%)	2,448 (99.3%)	1.20 (0.53–2.74)	0.66

* Values are shown as means (Standard deviation) or numbers (percent). mOR = refers to odds ratio from conditional logistic regression of matched case-control children in Global Enterics Multicenter Study (GEMS) study^40^; 95% CI = 95% confidence interval.

**Table 3:** Comparison of household hygiene indicators directly observed at the households of cases with moderate-to-severe diarrhea and their matched controls at a visit ∼60 days after enrollment in the Global Enterics Multicenter Study (GEMS)

	Case, *N* = 1,375	Control, *N* = 2,428	mOR (95% CI)	*P*
No handwashing station	11 (0.8%)	20 (0.8%)	Ref.	0.20
Handwashing station with water observed in house/yard	1,364 (99.2%)	2,408 (99.2%)	0.32 (0.06–1.80)	

Any cleansing materials observed in household where a handwashing station was present:				
Station with water only	9 (1.7%)	13 (1.5%)	Ref.	0.83
Station has water and a cleanser (soap, detergent or ash)	1,355 (99.3%)	2,395 (99.5%)	0.91 (0.37–2.20)	

Observed no cleansing material, soap/detergent, or ash:				
No cleanser	9 (0.7%)	13 (0.5%)	Ref	Ref
Soap or detergent	1,206 (88.4%)	2,115 (87.8%)	1.22 (0.44–3.36)	0.70
Any ash	655 (48.0%)	1,149 (47.7%)	0.80 (0.28–2.17)	0.63

Observed soap/detergent only versus ash only:				
Ash only (no soap)	149 (10.8%)	280 (11.5%)	Ref.	Ref.
Soap only (no ash)	700 (50.9%)	1,246 (51.3%)	0.91 (0.62–1.32)	0.61

* Values are shown as numbers (percent), mOR = refers to odds ratio from wealth-adjusted conditional logistic regression of matched case-control children in GEMS study^40^; 95% CI = 95% confidence interval.

**Table 4:** Distribution of observed handwashing materials in household handwashing areas according to wealth income quintile (WIQ)

WIQ	1st (poorest)	2nd	3rd	4th	5th (wealthiest)	Chi-square *P* value
All households						
Case	*N* = 287	*N* = 266	*N* = 276	*N* = 282	*N* = 263	0.7042
Control	*N* = 474	*N* = 492	*N* = 469	*N* = 498	*N* = 496	
Any soap	78.6%	84.3%	88.1%	91.7%	94.1%	*P* < 0.0001*
Case	77.0%	83.8%	88.4%	94.3%	96.2%	*P* = 0.85**
Control	79.5%	84.6%	87.8%	90.2%	92.9%	
Any ash	52.0%	49.7%	50.7%	47.9%	36.8%	*P* < 0.0001*
Case	54.0%	48.5%	50.7%	49.3%	35.4%	*P* = 0.50**
Control	50.8%	50.4%	50.7%	47.2%	37.5%	
Soap only	45.3%	48.4%	47.6%	51.5%	63.0%	*P* < 0.0001*
Case	43.2%	49.2%	48.0%	50.4%	64.6%	*P* = 0.98**
Control	46.6%	48.2%	47.3%	52.2%	62.1%	
Ash only	18.8%	13.9%	10.3%	7.8%	5.7%	*P* < 0.0001*
Case	20.2%	13.9%	10.5%	5.3%	3.8%	*P* = 0.10**
Control	17.9%	13.8%	10.2%	9.2%	6.7%	

* χ^2^ *P* value for trend in distribution of population using a handwashing material by wealth quintile.

** χ^2^ *P* value for trend in the distribution of cases and controls using a handwashing material by wealth quintile.

**Table 5:** Types of materials used for hygiene purposes and location of storage in households of caretakers in Mirzapur, Bangladesh

Materials	Timing	Place where kept
Bar soap	1. Handwashing	1. Inside the facility
	a. After defecation	2. Outside/beside the facility
	b. After cleaning bottom of baby who defecated	3. Near the handwashing area in a soil pot/in a poly-ethylene bag (usually tube well)
	c. Before feeding the child	
	d. After completing household tasks (contact with dirty things) including handling cow-dung	
	2. Bathing	
	3. Cleaning the child's bottom and hands	
	4. Laundry	
Detergent/washing powder	1. Handwashing	1. Outside/beside the facility
	a. After defecation	2. Near the handwashing area (usually tube well)
	2. Cleaning utensils	
	3. Laundry	
	4. Cleaning the sanitation facility	
Ash	1. Handwashing	1. Outside/beside the facility
	a. After defecation	2. Near the handwashing area (usually tube well)
	2. Cleaning utensils	
	3. Cleaning the sanitation facility	
Soil/dirt	1. Handwashing	1. (rub hands on) Ground in yard
	a. After defecation	2. (rub hands on) Ground outside the toilet
	b. After cleaning bottom of baby who defecated	
