# Supplementary material for: Association between Moderate-to-Severe Diarrhea in Young Children in the Global Enteric Multicenter Study (GEMS) and Types of Handwashing Materials Used by Caretakers in Mirzapur, Bangladesh
Source: Am J Trop Med Hyg. 2014 Jul 2;91(1):181–9. doi: 10.4269/ajtmh.13-0509 (PMC4080560; doi:10.4269/ajtmh.13-0509)
Supplement: Supplementary file 1 [file SD6.pdf]

Supplemental Table 1

Reported household ownership of 13 assets in each wealth index quintile for case households enrolled in the GEMS study in Mirzapur, Bangladesh

WIQ	1st (poorest)	2nd	3rd	4th	5th (wealthiest)
Number of sleeping rooms	1.4 (0.5)	1.8 (0.9)	2.0 (1.0)	2.8 (1.1)	3.8 (1.6)
Telephone	66 (22.8)	196 (72.3)	239 (85.7)	281 (97.9)	265 (99.3)
Electricity	42 (14.5)	109 (40.2)	207 (74.2)	272 (94.8)	263 (98.5)
Television	0 (0)	22 (8.1)	149 (53.4)	257 (89.6)	262 (98.1)
Agricultural land	70 (24.1)	157 (57.9)	166 (59.5)	227 (79.1)	217 (81.3)
Radio	5 (1.7)	22 (8.1)	69 (24.7)	109 (38.0)	161 (60.3)
Finished floor	1 (0.3)	9 (3.3)	23 (8.2)	43 (15.0)	158 (59.2)
Refrigerator	0 (0)	0 (0)	0 (0)	4 (1.4)	115 (43.1)
Bicycle	48 (16.6)	84 (31.0)	83 (29.8)	125 (43.6)	102 (38.2)
Scooter/motorcycle	0 (0)	1 (0.4)	9 (3.2)	6 (2.1)	63 (23.6)
Car	0 (0)	1 (0.4)	1 (0.4)	1 (0.4)	15 (5.6)
Boat	3 (1.0)	2 (0.7)	2 (0.7)	5 (1.7)	6 (2.3)
Cart	2 (0.7)	3 (1.1)	1 (0.4)	1 (0.4)	3 (1.1)
Total	290	271	279	287	267

*Number of sleeping rooms is shown as mean (standard deviation), and the rest are shown as number (percent) of households in that quintile who report owning that asset.
